# Repair of soft tissue and extensor tendon defects on the dorsum of the hand by transfer of dorsal foot flap and extensor digitorum brevis tendon in a 3-year-old child: A case report

**DOI:** 10.1097/MD.0000000000021837

**Published:** 2020-08-21

**Authors:** Heng Tian, Debiao Song, Hongjuan Jin, Quanzhe Liu, Yongheng Zhao, Xuejie Wang, Wenrui Qu, Rui Li

**Affiliations:** a Department of Hand Surgery, the Second Hospital of Jilin University, 218 Ziqiang Street, Changchun, Jilin; b Department of Emergency and Critical Medicine, The Second Hospital of Jilin University; c Department of Plastic and Reconstructive Surgery, The First Hospital of Jilin University, Changchun, Jilin; d Department of Hand and Foot Surgery, LinYin People's Hospital, Linyi, Shandong, P. R. China.

**Keywords:** case report, dorsal foot flap, extensor digitorum brevis tendon, extensor tendon defects, soft tissue

## Abstract

**Rationale:**

Repair of soft tissue defects on the dorsum of the hand with accompanying tendon defects is a challenging problem in clinical practice.

**Patient concerns:**

Here, we describe the case of a 3-year-old boy with a 1-week old soft tissue injury with infection due to a soft tissue defect on the dorsum of his right hand, and further describe its treatment.

**Diagnosis:**

A diagnosis of a soft tissue defect of the dorsum with extensor tendon defects in the fore, middle, ring, and little fingers of the right hand was made.

**Interventions:**

The defects were repaired using a dorsal foot flap combined with the extensor digitorum brevis tendon, under spinal anesthesia, and a small dose of the sedative phenobarbital (Lumina) was administered via pump injection after the surgery.

**Outcomes:**

The patient was followed-up for 6 months. The shape of the dorsal hand flap recovered satisfactorily and the skin color was almost normal. Protective sensation was restored and the tendon graft functioned well in vivo. Satisfactory outcomes were achieved in the flexion and extension of each finger.

**Lessons:**

This case study provides evidence that for soft tissue defects on the dorsum of the hand with tendon defects, 1-stage transfer of a dorsal foot flap with the extensor digitorum brevis tendon can be effective for recovery of appearance and extensor function. In case of infant patients, postoperative use of low-dose sedation can effectively reduce the risk of vascular crisis, thus promoting survival of the flap graft, and ensuring the success of the operation.

## 1 Introduction

Bicycle-spoke injuries of the hand in children are rarely reported.^[1,2]^ This type of injury produces severe compression or crush injuries to the soft tissues of the hand, often accompanied by exposure of bones, joints, tendons, and blood vessels.^[2]^ Improper treatment can easily cause disability and teratogenesis. With the growing demand for shape and functional reconstruction after hand injuries, traditional methods such as local and distant pedicled flaps have failed to yield satisfactory results. Rapid developments in microsurgical technology have attracted much attention to the use of free dorsal foot flaps for treating soft tissue defects on the dorsum of the hand.^[3]^ Considering its similar physiological structure, the free dorsal foot flap can be used to repair soft tissue defects on the dorsum of the hand.^[3]^ Good appearance, moderate texture, and satisfactory recovery of sensory function can be achieved after nerve joining.^[4]^ Moreover, the free dorsal foot flap with extensor digitorum brevis tendon can be used to repair extensor tendon defects.^[3]^ Due to a small vascular diameter, free skin flap transplantation is relatively risky in children and requires high-level anatomical and anastomosis technologies. Currently, few studies have reported free dorsal skin flap transplantation in children. Here, we report a case of using the dorsal foot flap in combination with the extensor digitorum brevis tendon to repair soft tissue and extensor tendon defects in the hand of a 3-year-old child, with satisfactory results. After the literature review, the child reported in our case was found to be the youngest patient to undergo successful dorsal foot flap with tendon transfer at stage I for dorsal hand defects.

This manuscript follows the Surgical CAse REport Guidelines,^[5]^ and written informed consent was obtained from the parents of the patient for the publication of this manuscript and the accompanying images.

## 2 Case report

**Figure 1:**
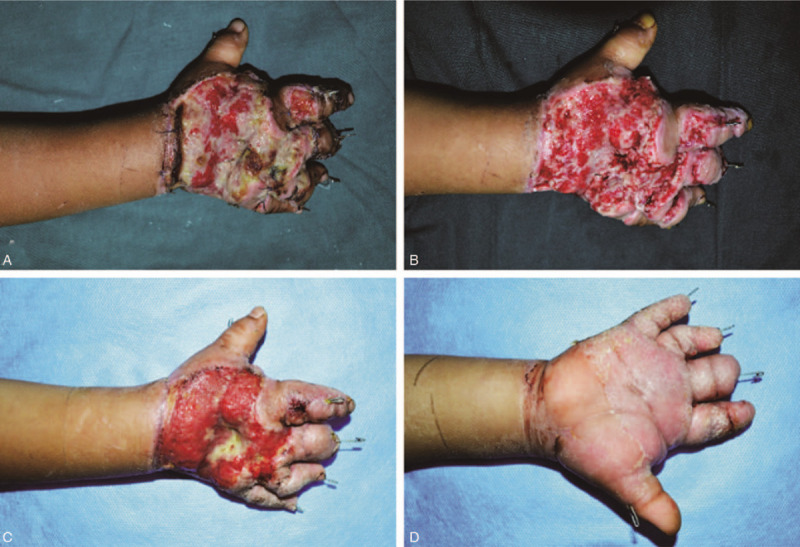
(A) The dorsum of the hand showed poor granulation tissue growth and an infected wound 1 wk after the injury; (B) The dorsum of the hand after using the VSD device indicated that the wound surface condition was improved 2 wk after the injury; (C, D) The dorsum and palm of the hand before surgery showed that the wound surface was ruddy, with granulation tissue growing well and uniformly, and there was no sign of infection. VSD = vacuum sealing drainage.

A 3-year-old boy was admitted to the hospital with a 1-week old soft tissue injury with accompanying infection on the dorsum of his right hand. The child's right hand was crushed by wheels, resulting in bicycle-spoke injuries 1 week before he was brought to the hospital. An external hospital diagnosed “metatarsal 2–5 fractures of the right hand, distal segmental mutilation of the right middle finger, soft tissue defect on the right dorsal hand (5 × 7 cm) with extensor tendon defects in the fore, middle, ring, and little fingers of the right hand.” Treatments at the other hospital included a reduction of metacarpal fractures with Kirschner wire internal fixation, stump repair of the right middle finger, and debridement of the dorsal hand. However, most of the extensor tendons of the right fore, middle, ring, and little fingers were crushed and lost. Postoperatively, granulation tissue growth was poor, and the wound was infected. To further repair the dorsal hand defects, the child was transferred to our hospital at 1-week post injury. Upon physical examination, the boy presented with the soft tissue defects of the right dorsal hand (5 × 7 cm) with extensor tendon defects in the fore, middle, ring, and little fingers of the right hand. Granulation and necrotic tissues were observed on the wound surface (Fig. 1A). The child underwent a reduction of the metacarpal fractures with a Kirschner wire internal fixation following stump repair of the right middle finger. X-ray examination showed that the fractures were well reduced with proper fixation of the Kirschner wire.

**Figure 2:**
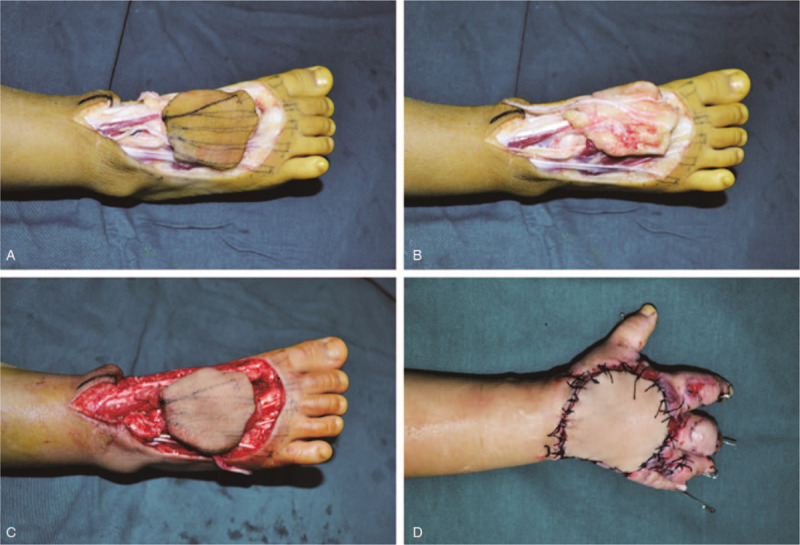
(A, B) Dorsal and ventral view of the free dorsal plantar artery combined with the extensor digitorum brevis tendon; (C). After loosening the tourniquet, the flap was ruddy in color and blood supply was good; (D) The appearance of the transferred dorsal foot flap combined with extensor digitorum brevis tendon on the dorsum of the hand.

Under general anesthesia, the upper limbs of the boy were wrapped with tourniquets. After routine surgical disinfection, the wound on the dorsal hand was thoroughly debrided and expanded to remove necrotic tissue followed by labeling of the damaged tendons (extensor tendons of the right fore, middle, ring, and little fingers). The dorsal hand defect was then covered with a vacuum sealing drainage (VSD) device fixed at the edge and was sealed with a semi-permeable membrane. Continuous suction using negative pressure was then performed. After 1 week, the VSD device was removed, and the wound was relieved (Fig. 1B). After 3 days of dressing changes (once a day), the wound surface before surgery was ruddy with well and uniformly growing granulation tissue and no sign of infections (Fig. 1C and D). Therefore, combined transfer of the dorsal foot flap and tendon at stage I was planned under general anesthesia (Fig. 2A–C).

First, the distal and proximal ends of the extensor tendon in each finger were separated from the dorsal hand, and the length of tendon defects was measured. The ipsilateral dorsal foot served as the donor. The axial lead of the flap was defined along the dorsal plantar artery. A composite flap graft was designed based on the size of the dorsal hand defect with the distal end to the toe web, the proximal end to the extensor retinaculum, and both sides to the 1st and 5th metatarsal bones. The dorsal plantar artery and its accompanying veins were exposed between the extensor hallucis longus and the extensor digitorum longus. The dorsal plantar artery was carefully separated up to the deep plantar branch and dorsal digital branch, followed by isolation of the superficial peroneal nerve up to the medial and middle dorsal cutaneous nerves. The nerves were ligated at the base of the 1st metatarsal bone space; the deep plantar branch and accompanying vein of the dorsal plantar artery were cut off. The flap was separated from the distal end to protect the tendon of the extensor digitorum longus. Intraoperative exploration of the extensor digitorum brevis tendon with complete aponeurosis was performed at the metatarsophalangeal joint. The flap was further cut off toward the proximal end. According to the length of the tendon defect, the extensor digitorum brevis tendon was correspondingly cut off at the proximal end. Moreover, the dorsal artery and its accompanying vein, as well as the medial dorsal cutaneous nerve of the superficial peroneal nerve, were used as needed.

**Figure 3:**
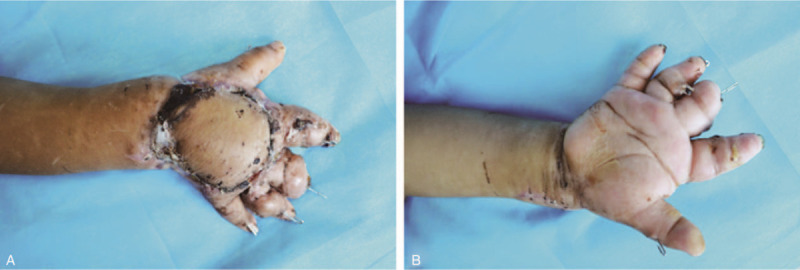
The transferred flap survived and showed movement of extensor muscles in each finger 2 wk after surgery.

The radial artery and its accompanying vein and superficial branch of the radial nerve were subsequently separated at the anatomical snuffbox of the wrist. The separated flap was placed on the wound surface of the dorsal hand, followed by anastomosis between the dorsal foot artery and its accompanying vein, and the radial artery and its accompanying vein, between the medial dorsal cutaneous nerve of the superficial peroneal nerve and the superficial branch of the radial nerve, and between the 2 ends of the extensor digitorum brevis tendon and the extensor digitorum tendon (Fig. 2D). Afterward, the tourniquet was released and the anastomosed blood vessels were observed to pulsate properly. The flap color and temperature gradually returned to normal, and the capillary response was normal. Finally, the mid-thickness skin flap of the ipsilateral thigh was used to cover the wound of the donor site. Postoperatively, the child's hand was fixed in a functional position with a plaster cast. In addition to conventional methods such as warming, fluid replacement, anti-infection, anti-spasmodic and anti-coagulant, and improved microcirculation, a small dose of phenobarbital (Lumina, 3 mg/kg)^[6,7]^ was administered via pump injection to keep the child in a lethargic state. The child had a wakeful response to command and could complain of the site of pain with a white value scored 3.^[8]^ Vital signs were closely monitored post operation. The flap survived till 2 weeks after the operation (Fig. 3A and B). X-ray examination at 6 weeks showed that the metacarpal fracture healed well. The Kirschner wire was removed and rehabilitation exercise for hand function was gradually performed. During the 6-month follow-up, the shape of the dorsal hand flap was satisfactory, and the skin color was nearly normal. Protective sensation was restored, and the tendon graft functioned well in vivo. Satisfactory outcomes were achieved in the flexion and extension of each finger. The donor site on the dorsum of the foot healed well without ptosis of the toes.

## 3 Discussion

Repair of soft tissue defects on the dorsum of the hand with accompanying tendon defects is a challenge in clinical practice. Conventional repair of such wounds requires skin flap transplantation at stage I with free tendon transfer as a stage II procedure. However, recovery of hand function can be delayed by a long treatment course, joint stiffness due to long-term immobilization, and tendon adhesion.^[9–11]^ With the development of microsurgical technology, the choice of a skin flap with tendon transfer for wound repair at stage I has become a treatment trend.^[12–14]^ Complete blood supply around the tendon in the composite flap results in rapid healing and mild adhesion after tendon transplantation, which helps restore the function of the finger.^[13]^ Ever since Cobbett successfully implemented toe-to-hand transfer in 1969, stage I repair with composite tissue grafts using microsurgical technology has become a focus of attention.^[15]^ In early days, tissue grafts mostly comprised single structures.^[16,17]^ However, with the improved understanding of vascular anatomy, use of composite tissue grafts has now become possible.^[18,19]^ In 1979, Taylor and Townsend reported the first dorsal foot flap with extensor tendon transfer in the treatment of a large area of skin and subcutaneous defects in the dorsal hand and achieved satisfactory results.^[20]^ Subsequently, the technique was applied to treat more complex dorsal hand injuries and achieved satisfactory results. After literature review, we found that the child reported in our case was the youngest patient to undergo successful dorsal foot flap with tendon transfer at stage I for dorsal hand defects.^[3,21,22]^

Good wound conditions are an important prerequisite for the survival of transplanted skin flaps. Upon admission, wounds are often accompanied by contamination to different extents. Debridement is often performed to improve wound conditions, but poor healing and susceptibility to infection are some of the problems that remain to be addressed. In the present case, the child was transferred to our hospital due to poor curative efficacy. The child was subjected to VSD after debridement to control the wound infection. In 1992, VSD technology was first used by Fleischmann in the early treatment of open fractures.^[23]^ Currently, it has been developed as a standard treatment mode for wounds, infections, and ulcers, and has also proven effective in treating soft tissue defects in the limbs of children.^[24]^ Compared to traditional debridement and dressing methods, VSD does not require multiple debridements and dressing changes. It can also promote the growth of granulation tissue, reduce wound infections, and create optimum wound conditions for subsequent treatments. It can also reduce painful stimulation and psychological trauma to children and helps increase their compliance with treatment.^[24,25]^

Many types of skin flaps have been used to repair skin defects on the dorsum of the hand. The inguinal flap is the most commonly used distant flap that functions as either a pedicled flap or a free flap to provide stable skin coverage. However, the flap shape is enlarged and bloated postoperatively, with no sensation. Two or more surgical repairs are required, resulting in a long-term treatment course, which causes patient discomfort due to postoperative immobilization in a specific position. Thus is, therefore, not used in children. The use of a flap with a dorsal interosseous artery can cause scars on the forearm and affect appearance. Besides, improper operations can easily damage the deep branch of the radial nerve, which can cause poor dorsiflexion of the thumb.^[26]^ Use of the flap based on the dorsal branch of the ulnar artery results in loss of sensation after surgery, and the color of the flap is significantly different from the skin of the dorsum of the hand; scars are formed on the forearm, which affects the appearance of the hand. Moreover, flaps with a limited rotation arc can only cover a small defect and are not suitable for large skin defects on the dorsal hand and loss of multiple tendons.^[27]^ However, use of the dorsal foot flap has the following advantages^[19]^:

(1) Free dorsal foot flap with long pedicles, constant vascular anatomy, small diameter variations, and sufficient tissue to be cut off, which meets the needs of various types of tissue defects on the dorsum of the hand;
(2) The thickness and texture of the dorsal foot flap are similar to those of the skin on the dorsum of the hand. No secondary surgery is needed to improve the appearance of the hand after flap transplantation;
(3) The superficial branch of the radial nerve is sutured with the medial and (or) middle dorsal cutaneous nerves from the superficial peroneal nerve to restore the sensory function of the flap graft;
(4) The flap can carry a multi-vascularized extensor digitorum brevis tendon to form a composite tissue flap, which can simultaneously repair the defect and restore the function of finger extension;
(5) The flap donor site is more concealed and aesthetically more acceptable.

The dorsal foot flap combined with extensor digitorum longus tendon has been used widely. In the present case, we used a modified dorsal foot flap combined with extensor digitorum brevis tendon transfer to repair soft tissue defects and tendon defects on the dorsal hand, avoiding damage to the extensor digitorum longus tendon and maximally protecting the function of toe extension in the foot.

Nursing after pediatric flap transplantation has always been a clinical difficulty. Mental retardation, large emotional fluctuations, poor pain tolerance, and postoperative forced posture can exacerbate fear and anxiety in children. This causes sympathetic nerve excitement and increased catecholamine secretion. Peripheral blood vessels are therefore constricted followed by poor blood flow, resulting in vascular crisis. Generally, this occurs in 72 hours after surgery, especially within 12 hours after surgery. Proper prevention and management of vascular crisis is key to ensure the survival of the flap graft. A combination of Demerol, Phenergan, and Thorazine, commonly referred as DPT, has been used for sedation and analgesia in pediatric patients for more than 30 years.^[28–30]^ A low-dose hibernation mixture has been reported to prevent vascular crisis after pediatric flap transplantation and replantation.^[31,32]^ Both chlorpromazine and promethazine are phenothiazine-type sedatives, that mainly act on the limbic system, reticular structure, and hypothalamus. These 2 sedatives can effectively reduce the symptoms of anxiety, tension, and fear in children, steady the children's emotions, and ensure good sleep, which can significantly reduce the occurrence of vascular crisis, and improve the survival rate of the flap graft.^[28,33,34]^ However, due to certain incidences of adverse reactions resulting from the hibernation mixture, it is necessary to carefully evaluate the children's condition before using DPT. DPT use is strictly prohibited in children with a history of epilepsy, central nervous system abnormality, or congenital heart disease. The phenobarbital used in the present case is a typical representative of long-acting barbiturates.^[6,7]^ It has a better sedative effect and a hypnotic effect. Use of low doses of phenobarbital cannot excessively inhibit the central nervous system. In addition, the use of sedatives should follow all guidelines related to deep sedation, and the electrocardiograms, breathing, blood pressure, and pulse of children should be closely monitored.^[35]^

## 4 Conclusion

For soft tissue defects on the dorsum of the hand with tendon defects, one-stage transfer of the dorsal foot flap along with extensor digitorum brevis tendon can yield satisfactory outcomes in appearance and extensor function. In special cases of infant patients, postoperative use of low-dose sedation can effectively reduce the risk of vascular crisis, promote the survival of the flap graft, and ensure the success of the operation.

## Author contributions

**Conceptualization:** Heng Tian.

**Investigation:** Hongjuan Jin, Quanzhe Liu.

**Methodology:** Yongheng Zhao, Xuejie Wang.

**Writing – original draft:** Debiao Song.

**Writing – review & editing:** Wenrui Qu, Rui Li.
